# Almost 40 Years of Tissue Engineering in Russia: Where Are We Now?

**DOI:** 10.3390/biomedicines8020025

**Published:** 2020-02-05

**Authors:** Elena Alpeeva, Yury Sukhanov, Ekaterina Vorotelyak

**Affiliations:** N.K. Koltzov Institute of Developmental Biology of Russian Academy of Sciences, Department of Cell Biology, Vavilov str. 26, 119334 Moscow, Russia; yuri.sukhanov@gmail.com (Y.S.); vorotelyak@yandex.ru (E.V.)

**Keywords:** regenerative medicine, tissue engineering, skin equivalent, wound healing, clinical trials, marketing authorization, legislation, state strategy of development

## Abstract

This review describes achievements of Russian cell-based regenerative medicine in different periods of time depending on the legislation and politics, and future prospects for its commercialization and wide application with an emphasis on products devised for skin regeneration. The world’s experience in tissue engineering began with the development of living skin equivalents, utilizing a biopolymer matrix and cells at the very beginning of the 1980s. During this period, the USSR kept abreast with the times and also conducted studies on skin wound healing, implementing modern cell techniques. However, there soon emerged a gap between scientific advancement and practical application. After the breakup of the USSR, there were no institutions that could implement scientific inventions into full-scale manufacturing for clinical application. At the same time, accumulating scientific and practical experience allowed for the maintenance of biomedical research and its readiness for market entry at present. Recently developed legislation opens up new opportunities in this field in Russia. There are a growing number of studies on the development of novel products for regenerative medicine, bringing hope for its rapid progress.

## 1. Introduction

Regenerative medicine is a modern and rapidly developing field of medical science, which is based on cell and molecular biology and gene engineering, and utilizes achievements and cutting-edge inventions of all related scientific and practical disciplines. The main goal of regenerative medicine is to restore the lost structure or functions of organs and tissues by replacing or repairing damaged cells, or stimulating the body’s own regenerative resources by generating biologically active molecules.

Tissue engineering is one of the fields that became a part of regenerative medicine at the time when the term “regenerative medicine” had not even come into general use. Tissue engineering utilizes living cells, biocompatible materials, and suitable biochemical (e.g., growth factors) and physical (e.g., cyclic mechanical loading) factors, as well as combinations thereof, to create tissue-like structures [1].

Howard Green’s team at Harvard Medical School laid down the basis for the development of tissue engineering when they showed, in 1975, the serial cultivation of strains of human epidermal keratinocytes [2] and made the first approaches for the reconstruction of skin epidermis using them [3]. In 1983, Eugene Bell, with colleagues, published a paper describing the fabrication of the first living skin equivalents, consisting of two components: a dermal equivalent made of fibroblasts in a collagen matrix and an epidermis that developed from keratinocytes “plated” on the dermal equivalent [4].

## 2. The History of Tissue Engineering in Russia

In the USSR, work on the reconstruction of tissues and organs using cell cultures began in the early 1980s. The first successful transplantations of cells derived from skin were performed in Moscow at the Burn Center of the Institute of Surgery, named after Vishnevsky, with the involvement of specialists from the Institute of Biomedical Problems and the Institute of Biological Physics of the USSR Academy of Sciences. In 1988, scientists from the Institute of Biological Physics published a monograph, describing their fundamental work on skin reconstruction using fibroblasts, keratinocytes, and an extracellular matrix [5]. In 1989–1990, scientists from the Institute of Surgery reported the application of cultured epithelium [6] and fibroblasts [7] for burn wounds treatment. Subsequently, almost all the leading burn centers in Russia were involved in the development of skin tissue engineering, including Kirov Military Medical Academy (St. Petersburg) and the medical scientific institute, named after Sklifosovsky (Moscow). There are reports of the successful clinical use of keratinocytes cultured according to Rheinwald and Green’s method, written by the scientists of the Military Medical Academy, in collaboration with the group of V. Terskikh of Koltzov Institute of Developmental Biology of Russian Academy of Sciences (Moscow) [8,9]. Along with studies in the field of cell cultures, polymer materials, to serve as carriers for cells, collagen films and gels, were developed. An important role in these studies played the Institute of Cytology of the USSR Academy of Sciences (St.Petersburg). Since the late 1980s, together with the Military Medical Academy, it was involved in the government program for “Biotechnological cultivation of human skin cells to treat critical and supercritical burns”. In 2005 the first company manufacturing tissue equivalents in Russia was called the Centre for Cell Technologies and was designed according to GMP standards—established on the basis of the Institute of Cytology—that had already developed a tissue equivalent (dermal equivalent) containing collagen I or fibrinogen matrix and human dermal fibroblasts, which was to be produced there.

During the period between 2000 and 2010, several Russian patents described living skin tissue equivalents for skin wound healing. In 2000, the joint group of scientists from the Institute of Cytology and the Military Medical Academy patented the method of isolation and subsequent cultivation of keratinocytes (e.g., allogenic) on porous polymer substrates such as polyethylene tetraflurate, polyimide or carboxymethyl cellulose [10]. In 2005, researchers from the Institute of Developmental Biology patented an active biological complex for organogenesis, which represented a multicomponent three-dimensional structure containing human allogeneic or autologous fibroblasts (embryonic or adult) and keratinocytes, and at least one layer of a biocompatible polymer (made of collagen / collagen containing fibronectin and / or laminin and different growth factors) in the form of a mesh matrix [11]. In 2008, the Centre for Cell Technologies patented the skin equivalent, which contained extracellular fibrin or collagen gel matrix proteins with human mesenchymal cells (fibroblasts / embryonic fibroblasts / bone marrow mesenchymal stem cells) inside it and keratinocytes selected to show stem cell characteristics on its surface [12]. Additionally, in 2008, the State Scientific Centre for Virology and Biotechnology “Vektor” (Novosibirsk) patented the invention for the replacement cell therapy combining a biopolymer carrier (collagen–chitosan film / a crumb of collagen-chitosan film / high-polymeric gel made of a 5% macrogol solution 1500) and human embryonic lung or myocutaneous tissue fibroblasts [13]. The efficacy of all these patented inventions and methods (summarized in Table 1) was proven by use on patients.

According to the report by the expert of the Scientific Centre for Expert Evaluation of Medicinal Products V. Merkulov, made in course of all-Russian conference “The real way from scientific investigations to drugs” in 2017, at least 16 technologies (including derivation, cultivation, storage, transportation and application) and products for organ and tissue reconstruction had been registered for clinical use by the Federal Service for Supervision in Healthcare by the end of 2011 (Table 2).

These techniques and products passed studies in the laboratory that were not equivalent to those that are required by contemporary international legislation, and in several cases, also limited clinical trials, but were used in clinical practice. Thus, their safety and efficacy was not sufficiently proven.

## 3. Skin Tissue Engineering in Russia in the Last Decade

Though skin tissue engineering was the first branch of tissue engineering to develop, soon it took a back seat and scientists all over the world, including Russia, switched to developing cell products for the regeneration of other tissues and organs. In the Russian Federation, scientists then worked on bone [14] and cartilage [15] equivalents and the treatment of defects and dysfunction of different organs such as the urethra [16,17], bladder [18], heart [19], central nervous system [20], liver [21], gut [22], pancreas [23], eye [24], teeth [25], hair follicle [26], etc., using implants made of different polymeric scaffolds populated with living cells (summarized in Table 3). Other techniques like transplantations of the decellularized matrix, cells or scaffolds alone, or scaffolds impregnated with active biological molecules, cells bearing modified genes or bearing gene constructs, or differentiated induced pluripotent stem cells (iPSCs), are also popular in Russia. In the thesis book of the III National Congress on Regenerative Medicine (2017), which is a very important scientific meeting for those who conduct studies in that field, there are several interesting reports on new skin tissue engineering techniques. For example, scientists from Kazan described the application of gene modified allogenic dermal fibroblasts with improved regenerative properties, which was made by the insertion of the human fibroblast growth factor FGF-2 to their genome for treatment of third-degree burns in rabbits [27]. A research group from Ekaterinburg showed the results of the clinical trial of the product made of biodegradable polylactide microspheres with autologous fibroblasts or MSCs seeded on their surface on 10 patients suffering from II type diabetes mellitus with neurotrophic ulcers of the lower limbs [28]. Additionally, investigators from Moscow reported on the treatment of patients with inherited epidermolysis bullosa by intradermal introduction of allogenic fibroblasts [29], and investigators from Nizhnij Novgorod described the development of a scaffold on the basis of collagen and plasma fibrin imitating the extracellular matrix that can be polymerized with adipose tissue derived MSCs or fibroblasts inside it [30].

To compare the above-described Russian trends with what is going on in the field of skin tissue engineering in the world, one can refer to the overviews of world advances in it, e.g., those made by Vig et al. [31].

## 4. Legislation and Marketing Authorization of Biomedical Products in Russia

In 2011, the federal law “On the Basics of Citizens’ Health Protection” [32] came into force, which did not include the term “medical technology”, to which regenerative medicine and its products and devices belonged in Russia at the time. Thus, clinical application of cell products, practically speaking, became illegal for a period of time, until finally, in 2016, the federal law “On Biomedical Cell Products (BMCPs)” established the rules for introducing cell products into the market based on scaling technologies [33]. The law primarily only came into force in 2017 and several of its bylaws, related to the licensing of products, etc., began to work in 2018–2019. At the present, Russian legislation does not define the term “hospital manufacturing” and it is assumed that personified autologous products have to be subjected to the same authorization procedure as products for widespread use (“off the shelf”). At the same time, the question of the use of minimally manipulated cell products remains open. In this segment of the market, there is no legislation, either prohibitive or permissive.

From 2017, clinical trials of the BMCPs have to be conducted in accordance with the order of the Ministry of Health [34] and the State Standard “Good Clinical Practice” [35]. Clinical trials can only be conducted in centers that gained permission by the Ministry. Their accreditation is going on, and by now, there are only 35 centers on the list all over Russia. At the end of 2017, the expert of the Scientific Centre for Expert Evaluation of Medicinal Products A. Tikhomirova and her colleagues found 18 clinical trials of Russian cell products on www.ClinicalTrials.gov (using the word combinations “cell therapy” and “stem cell”), of which two were completed, one stopped, and others would have been completed in 2017–2020 [36]. Only one of them was devoted to the application of skin equivalents. Now we could only find about four trials, three of which with the status “unknown”, and one was completed. This shows the drawbacks of legislation and freezing of commerce intentions.

## 5. Cell Banking in Russia

At the same time, it should be mentioned that cell banking in the Russian Federation, which is a cornerstone of all cell technologies, also had no direct legislation for a long time. Originally banking of cell lines was mainly carried out by scientific centers for scientific research. The first collection of vertebrate cells, which is now the biggest scientific collection in Russia, was established in 1978 by the Institute of Cytology. However, these cell collections were also the first to be used for development of medical products. During the 1990s and the beginning of the 2000s, it was a difficult period when, in the absence of financing, a lot of cell line specimens were lost. Now, there are many small and rather big cell banks in different scientific centers, laboratories and hospitals. With the development of regenerative medicine, commercial and private banks of umbilical blood and MSCs from different tissues began to emerge. There are big commercial umbilical cord stem cell banks such as “Trans-Technologii” (https://trans-t.ru/) in St.Petersburg, “Hemabank” (https://gemabank.ru/) and “CryoCenter” (https://www.cryocenter.ru/tech) in Moscow and also several banks of this kind in budgetary organizations (e.g., in Moscow and Orenburg). Pokrovsky bank of stem cells (http://stemcellbank.spb.ru) not only collects and stores cells from umbilical cord blood but also develops cell technologies for the treatment of allergies, burns, trophic ulcers, lower limbs ischemia and for use in stomatology, orthopedics, traumatology.

After the federal law “On Biomedical Cell Products” was adopted, the “Requirements for the organization and activity of “biobanks” (cell banks) and the rules for storage of the biological materials, cells for cell line preparation, cell lines for the production of BMCPs” were developed and approved by the order [37]. This was the first regulatory act relating directly to cell lines (rather than umbilical cord blood or bone marrow) containing mandatory legislative rules for staff, premises, equipment and documentation for storage of the biological objects and BMCPs and requirements for the quality control system.

But still the question of quality control when cell banks provide or store material for development of medical products is very serious and needs elaboration of laws and establishment of controlling bodies in order to provide safety of patients.

## 6. Perspectives

Though now market share of Russia in the world biomedical technologies is less than 1% [38] regenerative medicine and biodegradable materials are among the most significant and promising fields of medicine in the “Long-term forecast of the scientific and technological development of Russia for the period till 2030” provided in 2013 [39].

The recently established State Scientific and Technical Program for the Development of Gene Technology in the Russian Federation in 2019–2027 [40], among others, has the goal of developing no less than 20 gene therapy and biomedical products containing gene modified cell lines that would pass preclinical trials by 2027. Table 4 summarizes the main regulatory acts that determined the development of regenerative medicine in Russia in the last decade.

Research and pilot studies that have been conducted in Russia lately have the tendency to switch to the application of pure MSCs suspensions intravenously or exactly to the place of injury and even with minimal manipulation, which is well seen.

At the same time, creating BMCPs requires huge investments and is associated with high economic risk. Regenerative medicine in Russia urgently needs state support and stimulation of commercialization. The interaction of research teams with clinical specialists and communities, manufacturing companies and regulatory bodies is very important. It is necessary to pay more attention to the expert assessment of potentially commercialized investigations carried out through state foundation financing, to involve business representatives as potential sources of financing and representatives of regulatory bodies in estimating the potential outcomes of investments. Additionally, this is a measure compliant with the “Strategy of the development of medical science in the Russian Federation for the period till 2025” [41], according to which it is necessary to increase opportunities for the commercialization of already developed innovative products with guaranteed purchases by the state. The first application for licensing of the industrial manufacturing of BMCPs was submitted by the scientific and production company Generium in 2018, but it has not gained approval yet. While the Center for Cell Technologies is still at the stage of GMP approval and has not even submitted the application for manufacture licensing.

In 2017 the Ministry of High Education and Sciences authorized the project aimed at conducting preclinical trials and phase I and II clinical trials of two BMCPs for the treatment of burns and chronic wounds developed by the Institute of Developmental Biology. A partner biomedical enterprise Acrus BioMed LLC invested in the construction of the production site. Clinical trials are expected to be completed in 2021 and the first two BMCPs will hopefully enter the market.

## 7. Conclusions

In Russia, there has been enough research and investigations in the field of regenerative medicine. Nevertheless, in the last decade, while bioengineering companies in the USA, Europe and Asia conducted full-scale clinical trials of their products, obtained marketing authorizations for them, manufactured and marketed them, due to the lack of legislation, Russian scientific centers were developing tissue engineering technologies that were only used by small private clinics and were not permitted for use in the official healthcare system. Now, legislation is being developed, although it is still weak and does not cover all the essential questions. The adoption of the federal law “On Biomedical Cell Products” gave rise to the extensive work in this direction and we hope that soon all the main drawbacks in legislation will be eliminated. Biomedicine is included in all the main state programs of development, BMCPs production sites are being established now and the government is working on the problem of attracting investments into this field. We expect that soon at least two skin wound healing BMCPs designed and produced with full compliance with the new legislation will obtain marketing authorization and enter the market.

## Figures and Tables

**Table 1:** Different types and composition of skin tissue equivalents patented in Russia in the 2000s.

Extracellular Matrix	Cell Component
polyethylene tetraflurate/polyimide/carboxymethyl cellulose	keratinocytes
collagen/collagen containing fibronectin and/or laminin (and different growth factors)	keratinocytes and fibroblasts (embryonic or adult)
fibrin/collagen	keratinocytes and fibroblasts (embryonic or adult)/bone marrow mesenchymal stem cells
collagen-chitosan film/a crumb of collagen-chitosan film/high-polymeric gel made of a 5% macrogol solution 1500	embryonic lung/myocutaneous tissue fibroblasts

**Table 2:** Technologies and products registered in Russia by the end of 2011 to correct tissue defects.

Disease/Tissue to Be Restored	Source of the Cells	Matrix
skin and other tissues	allogenic and autologous fibroblasts	− *
skin and other tissues	dermal fibroblasts	collagen I/fibrinogen
heart muscle	autologous bone marrow MSCs differentiated into cardiomyoblasts	
bone defects of the upper and lower jaw	autologous MSCs differentiated in osteogenic direction	matrix
cartilage tissue	autologous chondroblasts	three-dimensional gelatine matrix
chronic lower limb ischemia	MSCs	− *
recessions and mucosal deficiency in the area of teeth and dental implants	fibroblasts of the oral mucosa	− *
ulcerative colitis and Crohn’s disease	MSCs	− *
tuberculosis	MSCs	− *

* These hyphens mean that in these products no matrix was used.

**Table 3:** Examples of studies on regeneration of different organs and tissues in Russia in the 2010s.

Reference	Organ/Tissue to Be Regenerated	What Was Investigated or Developed in the Study	Components Used
[14]	bone	the reparative osteogenesis in the rabbit mandible defect in the early stages of healing	polylactide scaffolds (modified by collagen or fibrin solutions) and bone marrow MSCs
[15]	cartilage	a method of chondroitinsulfate depositing on polylactic scaffolds – in vitro study	polylactic scaffolds covered by chondroitinsulfate for better cell adhesion; newborn rabbit bone marrow MSCs
[16]	urethra	tissue equivalent for the closure of extended urethral defects (rabbits)	collagen gel with embedded fibroblasts and epidermal keratinocytes grown on its surface; lavsan-mesh endoprosthesis as the framework
[17]	urethra	transdifferentiation of epidermal keratinocytes in vivo in a model of the recovery of urethral injuries in rabbits	skin keratinocytes
[18]	bladder	a potential approach for bladder tissue engineering in a model of partial bladder wall cystectomy in rabbits	a poly-L-lactide/silk fibroin bilayer scaffold seeded with allogenic bone marrow stromal cells
[19]	heart	persistence of rat cardiosphere-derived cells after allogeneic transplantation into the peri-infarction zone by intramyocardial injection in rabbits	myocard cells (with luciferase reporter system) in the form of cardiospheres in suspension in a culture medium or in platelet rich plasma
[20]	central nervous system	the possibilities of transplantation of autologous neural progenitor cells from C57BL/6 mouse nasal olfactory lamina propria during a reconstructive operation after an open traumatic brain injury	neural progenitor cells from nasal olfactory lamina propria in the hydrogel based on low-, medium-, and high-molecular weight hyaluronic acid
[21]	liver	the effectiveness of cell therapy for the correction of chronic liver failure in chronic fibrotic liver damage in Wistar rats	suspension of allogenic liver cells or allogenic liver cells coculted with mesenchymal bone marrow stromal cells in biodegradable biopolymer gel resembling collagen (Sphero^®^GEL) used as matrix
[22]	gut	regenerative properties of fibroin implant vitalized with allogenic bone marrow MSCs using the experimental model of rat jejunum wall damage	MSCs seeded on fibroin fiber scaffold
[23]	pancreas	influence of intraperitoneal injection of tissue engineered constructs of pancreas on experimental diabetes mellitus in Wistar rats	floating insular-like cultures derived from pancreas of newborn rabbits cultured in presence of heterogeneous biopolymer hydrogel containing collagen
[24]	eye	in vitro properties of different polylactide matrices made for transplantation of cultured limbal stem cells to eliminate a limbal deficiency	polylactide-glycolide, polylactide-caprolactone, poly-ɛ-caprolactone; human and rabbit limbal stem cells, human corneal epithelial cell line
[25]	teeth	regeneration of dental pulp tissue using pulpal autologous MSCs and platelet-rich plasma transplanted in pulp chamber of miniature pigs after pulp removal	mesenchymal stromal cells from pulp of molars in combination with fibrin clot
[26]	hair follicle	hair germ model in vitro	human postnatal dermal papilla cells and skin epidermal keratinocytes cocultured in a hanging drop to develop an artificial hair follicle germ

**Table 4:** Basic regulatory acts specifying development of regenerative medicine in Russia currently.

Regulatory Act’s Number	Basic Effective Date	Regulatory Act’s Name
The state standard R 52379–2005	01.04.2006	“Good Clinical Practice”
The federal law N 323-FZ	21.11.2011	“On the Basics of Citizens’ Health Protection”
The federal law N 180-FZ	01.01.2017	“On Biomedical Cell Products”
The order of the Ministry of Health of the Russian Federation N 669n	13.11.2017	“On the approval of the Rules of Good Clinical Practice of Biomedical Cell Products”
The order of the Ministry of Health of the Russian Federation N 842n	13.04.2018	“On the approval of the requirements for the organization and activity of biobanks and the rules for storage of the biological materials, cells for cell line preparation, cell lines for the production of biomedical cell products”
The Government resolution N 479	22.04.2019	“On the approval of the State Scientific and Technical Program for the Development of Gene Technology in the Russian Federation in 2019–2027”
